# Methylmercury toxic mechanism related to protein degradation and chemokine transcription

**DOI:** 10.1186/s12199-020-00868-3

**Published:** 2020-07-17

**Authors:** Jin-Yong Lee, Gi-Wook Hwang, Akira Naganuma, Masahiko Satoh

**Affiliations:** 1 Laboratory of Pharmaceutical Health Sciences, School of Pharmacy, Aichi Gakuin University, 1-100 Kusumoto-cho, Chikusa-ku, Nagoya, 464-8650 Japan; 2 Laboratory of Environmental and Health Sciences, Faculty of Pharmaceutical Sciences, Tohoku Medical and Pharmaceutical University, Sendai, 981-8558 Japan; 3 Laboratory of Molecular and Biochemical Toxicology, Graduate School of Pharmaceutical Sciences, Tohoku University, Sendai, 980-8578 Japan

**Keywords:** Methylmercury, Ubiquitin-proteasome pathway, Pyruvate, Mitochondria, Chemokines

## Abstract

Methylmercury is an environmental pollutant that causes neurotoxicity. Recent studies have reported that the ubiquitin-proteasome system is involved in defense against methylmercury toxicity through the degradation of proteins synthesizing the pyruvate. Mitochondrial accumulation of pyruvate can enhance methylmercury toxicity. In addition, methylmercury exposure induces several immune-related chemokines, specifically in the brain, and may cause neurotoxicity. This summary highlights several molecular mechanisms of methylmercury-induced neurotoxicity.

## Introduction

Methylmercury is a toxic metal that causes severe central nervous system disorders such as Minamata disease [1] and is produced by the biomethylation of inorganic mercury by microorganisms [2]. Methylmercury enters the aquatic food chain and accumulates in carnivorous fish [2, 3]. Levels of methylmercury in seawater have been estimated at 10.7–99.1 pg/L and those in swordfish at 249–1187 μg/kg [3]. The biomethylation and bioaccumulation of methylmercury result in human exposure through consumption of fish, and high intake can exert health risks, especially to the developing fetus [4, 5].

More than 90% of ingested methylmercury is absorbed from the gastrointestinal tract [4], and approximately 10% reaches the brain [4, 6]. Methylmercury can also cross the placental barrier, and methylmercury levels in the fetal brain can reach 5–7 times higher than those in maternal blood [7], which indicates the high health risk to the fetus. The main adverse effect of methylmercury exposure is neurotoxicity [8], and the clinical manifestation of methylmercury poisoning includes paresthesia, ataxia, vision and hearing loss, tremors, and spasticity [9, 10].

The human pharmacokinetics and pharmacodynamics of methylmercury have been elucidated, and several molecular events, such as the generation of reactive oxygen species and disruption of calcium homeostasis, can be induced by methylmercury [11, 12]. Several intracellular pathways of methylmercury toxicity have been identified; this brief review discusses for the current understanding of the molecular mechanisms related to methylmercury-induced neurotoxicity.

## The ubiquitin-proteasome system and mitochondrial function

Budding yeast (*Saccharomyces cerevisiae*) is a unicellular eukaryote, and many of its gene products have similar functions to those of mammals [13]. *CDC34* has been identified as a gene that confers resistance to methylmercury in yeast cells [14, 15]. *CDC34* encodes the Cdc34 protein, a ubiquitin-conjugating enzyme, which is involved in the ubiquitin-proteasome protein degradation system [16]. Overexpression of Cdc34 in human cells has been shown to result in significant resistance to methylmercury [15], and this protective effect is suppressed by the inhibition of proteasome activity [15]. These findings suggest that methylmercury induces cellular accumulation of certain proteins and that this accumulation is toxic; further, these proteins are degraded via the ubiquitin-proteasome system.

Cdc34 is an E2 enzyme of the ubiquitin-proteasome system and interacts with the Skp1/Cdc53/F-box protein (SCF) E3 complex [16]. In budding yeast, 17 F-box proteins bind to substrate proteins degraded by the ubiquitin-proteasome system [17], and each F-box protein has a specific substrate in selecting proteins to be degraded. Overexpression of Hrt3 and of Ylr224w (Ucc1), two F-box proteins, has been shown to confer resistance to methylmercury in yeast cells [18]. In addition, yeast cells overexpressing Hrt3 and Ucc1 are not resistant to methylmercury in the presence of a proteasome inhibitor [18]. This indicates that the substrates recognized by Hrt3 or Ucc1 may include proteins that are involved in methylmercury toxicity and degraded by the proteasome. Therefore, the identification of these substrate proteins would assist in clarifying the molecular mechanism underlying methylmercury toxicity.

**Fig. 1:**
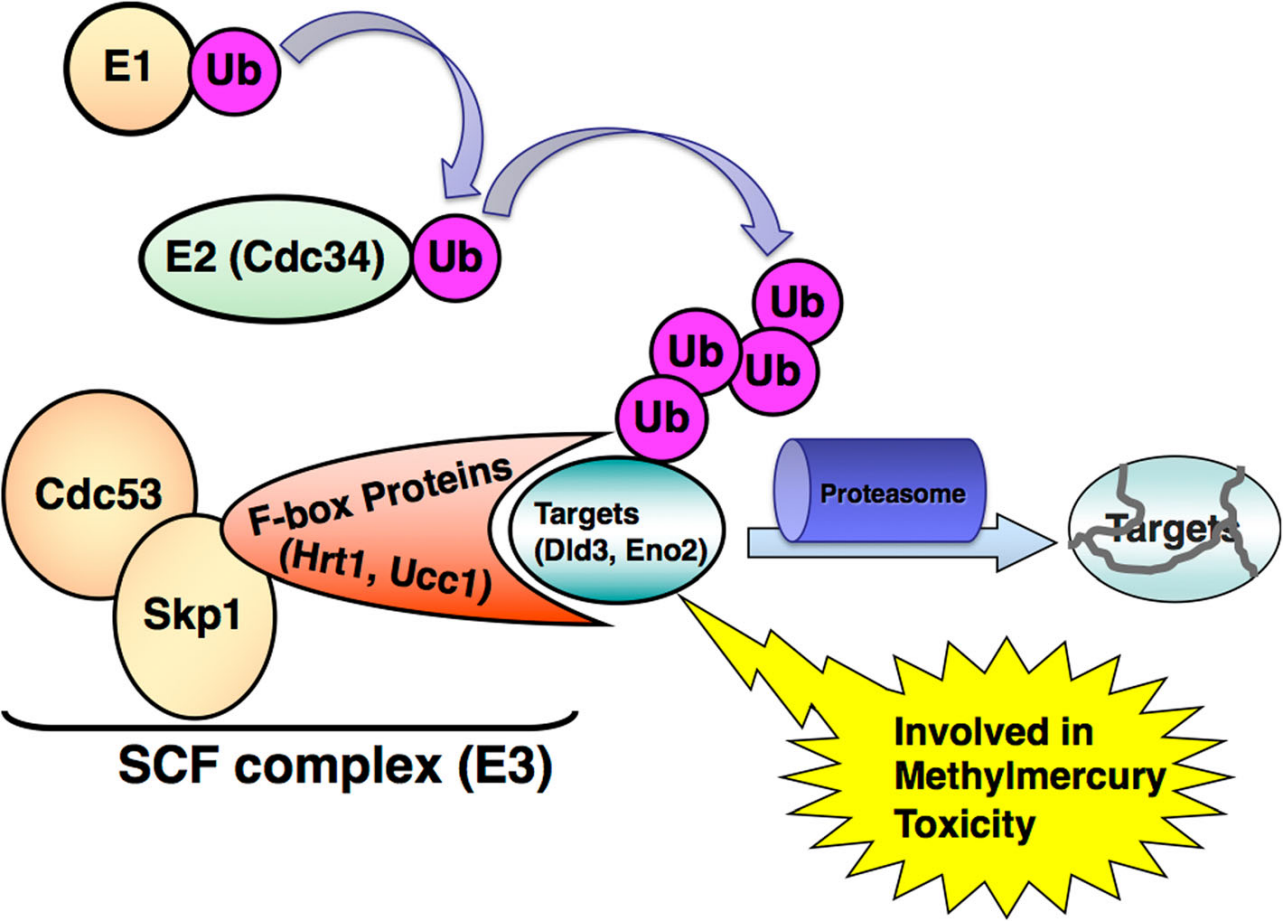
The ubiquitin-proteasome system involved in methylmercury toxicity. The target proteins, Dld3 and Eno2, respectively specific to Hrt1 and Ucc1 enhance methylmercury toxicity, unless they are degraded by ubiquitin-proteasome system. E1, ubiquitin activating enzyme; E2, ubiquitin conjugating enzyme; E3, ubiquitin ligase; Ub, ubiquitin

Yeast two-hybrid screening identified Dld3 as a substrate ubiquitinated by Hrt3 and Eno2 as a substrate ubiquitinated by Ucc1 [19]. Yeast cells overexpressing Dld3 and Eno2 exhibited higher sensitivity to methylmercury [19], indicating that Hrt3 and Ucc1 are involved in promoting proteasomal degradation by ubiquitination of Dld3 and Eno2 (Fig. 1). Dld3 is involved in the conversion of d-lactose to pyruvate [20] and Eno2 is involved in the glycolytic system [21], a metabolic pathway by which glucose is converted through several stages of reactions to pyruvate [22]. Yeast cells with overexpression of Dld3 and Eno2 showed higher sensitivity to methylmercury [19]. These suggest that an increase in the synthesis of intracellular pyruvate is one pathway of methylmercury toxicity. We previously demonstrated that the addition of non-toxic concentrations of pyruvate enhances the sensitivity of yeast and human neuroblastoma cells to methylmercury [23], suggesting that accumulation of Dld3 and Eno2, substrate proteins of the SCF complex, increases methylmercury toxicity, as does pyruvate, the metabolic product of glycolysis.

**Fig. 2:**
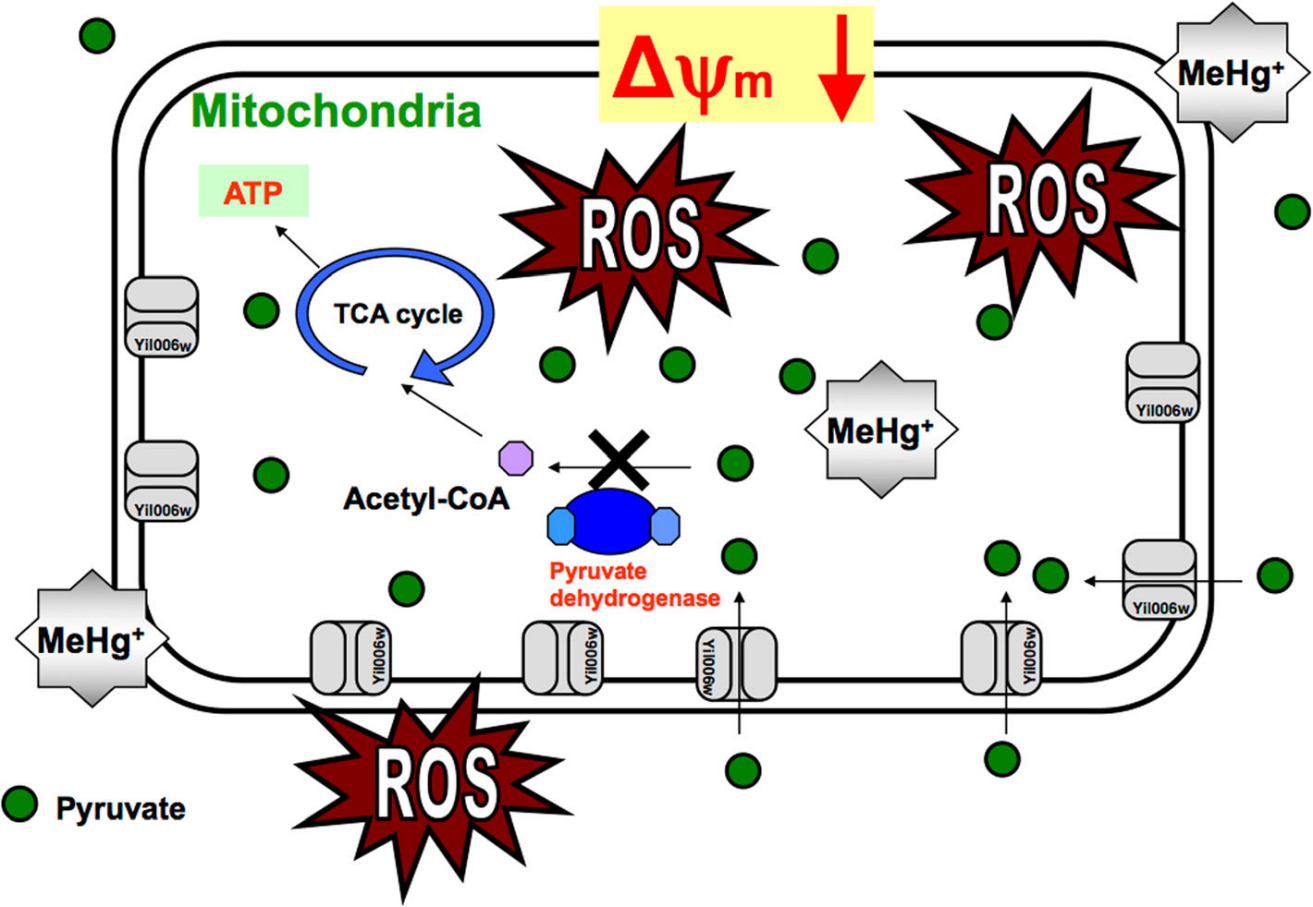
The scheme of methylmercury toxic mechanism by the pyruvate transportation into mitochondria. MeHg^+^, methylmercury; ΔΨm, mitochondrial membrane potential; ROS, reactive oxygen species

The mechanism causing pyruvate-induced methylmercury toxicity has also been examined. In yeast cells that have little pyruvate decarboxylase activity involved in the production of acetyl-CoA from pyruvate, the effect of pyruvate on methylmercury toxicity was significantly increased [23]. This result suggested that methylmercury promoted the transport of pyruvate into mitochondria and that the increased pyruvate concentrations in mitochondria were involved in intensifying the toxicity of methylmercury without being converted to acetyl-CoA. Furthermore, in human neuroblastoma cells, methylmercury treatment alone decreased the mitochondrial membrane potential, and the addition of pyruvate led to a further significant decrease. In addition, treatment with *N*-acetylcysteine (an antioxidant) significantly alleviated the toxicity of methylmercury and significantly inhibited the intensification of methylmercury toxicity by pyruvate [23]. These data indicate that methylmercury increases mitochondrial pyruvate levels, leading to mitochondrial dysfunction and the generation of reactive oxygen species (Fig. 2).

## Chemokines in methylmercury toxicity

The precise mechanism of methylmercury-induced neuronal disorders is unclear. Our research group previously found that methylmercury treatment altered the expression levels of genes in the murine cerebellum, upregulating the expression of 21 genes and downregulating that of 11 genes [24]. Increased expression was observed for several genes encoding the chemokines Ccl2, Ccl4, Ccl7, Ccl9, and Ccl12. Chemokines have been hypothesized to be involved in physiological synaptic signal transmission and developmental processes in the central nervous system [25], and their roles in methylmercury toxicity should therefore be investigated. We found that in the murine cerebrum, mRNA levels of these five chemokines increased significantly in response to methylmercury, and a similar response was observed in the kidney, with the exception of *Ccl4* expression. No significant effect on chemokines was observed in the liver and spleen [26]. Our study of changes in chemokine gene expression in the murine cerebellum, cerebrum, kidney, liver, and spleen found that methylmercury-induced upregulation of *Ccl3* and *Ccl4* expression [27], implying a specificity in methylmercury toxicity to the central nervous system. We also found that methylmercury increased *Ccl2* expression in human 1321 N1 astrocytes and elevated nuclear levels of the NF-κB p65 subunit; overexpression of *CCL2* was inhibited by suppressing p65 expression using RNA interference [28]. More recently, we examined the transcriptional regulatory mechanism that induces *Ccl4* expression in C17.2 mouse neural stem cells, and found that methylmercury stimulated the region upstream of the transcription start site and increased nuclear levels of serum response factor (SRF) and the amount bound to the *Ccl4* gene promoter [29]. We also confirmed that methylmercury activated p38 and ERK, which are a part of the mitogen-activated protein kinase pathway, and these activations were involved in the induction of *Ccl4* expression [29].

## Conclusions

We summarized the known molecular mechanisms of methylmercury-induced neurotoxicity, including pyruvate. The findings suggest that the normal function of the ubiquitin-proteasome system in regulating pyruvate-promoting proteins may influence methylmercury toxicity. Glucose is the main energy source of the mammalian brain, and after conversion to pyruvate is used for ATP production [30]. Pyruvate accumulation in mitochondria may increase methylmercury toxicity.

Chemokines mediate inflammation in various tissues, including the brain and kidneys [31, 32]. Therefore, it is reasonable to postulate that chemokines are involved in the pathway mediating methylmercury toxicity. Chemokines may function as signaling molecules in the CNS [33]. Several chemokines may be specific to methylmercury-induced disorders of the central nervous system.

Our research group recently reported several factors involved in the molecular pathways of cadmium, as well as methylmercury [34, 35]. Such mechanistic studies in other metals may contribute to our understanding of the toxic mechanism of toxic metals. Further studies are very expected to elucidate the precise molecular mechanism of toxic heavy metals.

## Data Availability

Not applicable.
